# The Joint Mechanical Function and Control of the Front Leg During Cricket Fast Bowling: A 3D Motion Analysis Study

**DOI:** 10.3390/s26030902

**Published:** 2026-01-29

**Authors:** René E. D. Ferdinands, Peter J. Sinclair, Max C. Stuelcken, Andrew J. Greene

**Affiliations:** 1 Discipline of Exercise and Sport Science, Faculty of Medicine and Health, University of Sydney, Sydney, NSW 2006, Australia; 2 School of Health, University of Sunshine Coast, Sippy Downs, QLD 4556, Australia; 3 School of Life and Health Sciences, Centre for Integrated Research in Life and Health Sciences, University of Roehampton, London SW15 5PJ, UK

**Keywords:** cricket fast bowling, front leg mechanics, joint power, angular impulse, eccentric control, lower limb kinetics, hip and knee biomechanics, motion analysis, run-up speed, ball release velocity

## Abstract

**Highlights:**

**What are the main findings?**

- Front leg motion in fast bowling is dominated by eccentric control with negligible concentric power at the hip and knee; knee extension at ball release reflects whole-body coordination rather than local torque generation.
- Run-up speed is the strongest predictor of ball release speed, while knee angle at front foot contact has no significant influence.

**What are the implications of the main findings?**

- Isolated hip or knee strength training is unlikely to modify front leg mechanics; coaching should prioritise system-level coordination, eccentric control, and whole-body momentum transfer.
- Flexor-based and flexor-braced techniques are mechanically viable under high impact forces, whereas enforcing a fully braced knee is often unrealistic.

**Abstract:**

Cricket fast bowlers rely on the front leg as a mechanical lever during front foot contact, yet the underlying mechanisms that govern front leg behaviour remain unclear. This study examined front leg mechanics in 18 junior fast bowlers (17.2 ± 1.7 years) using a 14-camera 3D motion capture system and force platforms. Joint power and angular impulse analyses were performed to quantify hip and knee extension–flexion mechanics from front foot contact to ball release, enabling the classification of joint function as active (concentric), controlled (eccentric), or negligible. Power and angular impulse profiles revealed that front leg motion was dominated by controlled (eccentric) power at both the hip and knee, indicating that the regulation of knee angle occurred primarily through eccentric braking rather than concentric quadriceps extension. These findings suggest that achieving a “braced leg” position via isolated knee extensor strengthening may be ineffective. To evaluate whether kinematics and anthropometry contributed to performance, a multiple linear regression model was used. Run-up speed at back foot contact emerged as the strongest predictor of ball speed, whereas knee angle at front foot contact showed only a small and non-significant effect. Overall, the results indicate that front leg behaviour reflects coordinated whole-body dynamics, and performance interventions should prioritise momentum generation and timing across the kinetic chain rather than isolated joint actions.

## 1. Introduction

Fast bowlers in cricket play a pivotal role in matches. They are required to release the ball at high speeds of more than 37.5 m s^−1^ for extended periods, typically 2–3 spells of 6–8 overs in the longer forms of the game (first-class and test matches) and 10 overs in one-day matches [1,2]. To generate high ball speeds, the bowler relies on a run-up, gradually building up the body’s centre of mass velocity in a manner akin to that of javelin throwing [3,4]. This motion culminates at front contact at the end of the delivery stride, when the centre mass decelerates [5]. During the front foot contact phase, the bowling arm accelerates, during which time the front leg acts as a lever over which the upper body segments rotate, transferring kinetic energy to the bowling hand via a general proximal-to-distal sequence [6] in accordance with the kinetic link principle.

In their review of the existing literature, Bartlett [4] reported the existence of three types of front leg action with respect to changes in knee extension–flexion angle. Portus [7] extended this classification, identifying four front leg techniques based on discrete patterns of knee extension–flexion angle from front foot contact to ball release: flexor (10° or more knee flexion followed by less than 10° knee extension); flexor–extender (knee flexion and extension of 10° or more); extender (less than 10° knee flexion followed by 10° or more knee extension); and constant brace (less than 10° knee flexion or 10° extension). In a sample of 42 bowlers, these researchers also found that the largest proportion of the bowling sample (59.5%) favoured the flexor technique, followed by the constant brace (21.4%), extender (9.5%), and flexor–extender (9.5%).

Some biomechanics researchers have reported a relationship between the knee extension angle of the front leg and ball release speed [8,9,10,11,12,13,14], suggesting that bowlers adapt their technique to extend the knee during front foot contact actively. However, these studies should be considered preliminary, as they recruited relatively small sample sizes, finding only weak to moderate relationships between front leg knee angle and ball speed. Kiely et al. [15] found a small to trivial relationship between front leg knee angle at front foot contact and ball release in a sample of high-performance bowlers. A meta-analysis by Ramachandran et al. [16] on the characteristics that influence ball speed showed a moderate correlation between the front leg knee angle at ball release and ball speed (r = 0.40). Other studies, including those by Stockhill and Bartlett [11] and Portus et al. [7], did not find a significant relationship between front leg knee extension angle and ball release speed. In contrast, a planar 16-segment whole-body torque-driven simulation model of fast bowling showed that the extension of the ankle and knee in the front leg was more pronounced compared to the matched simulation [17]. These findings suggest that statistical outcomes may be confounded on a broader scale owing to the intricacies and inherent inter-individual variability associated with pace bowling [18].

Although the relationship between knee extension and ball speed remains inconclusive, coaches often emphasise bowling with an extended front knee [19,20,21,22,23]. Commonly referred to as the “braced” front knee technique, coaching programmes often advocate a rigid, extended front knee during delivery as part of an optimum fast bowling model [24]. The flexor technique is thought to reduce release height and dissipate energy from the upper body [25]. A more favourable alternative is the flexor–extender technique, where the bowler flexes the knee upon initial front foot contact to attenuate the impact forces before extending the knee to lengthen the front leg lever. This technique has been proposed as mechanically advantageous, though it appears uncommon in practice [7,14].

Despite this longstanding coaching emphasis on the front leg technique, relatively little research has investigated the biomechanical mechanisms that enable or constrain knee extension during fast bowling. Existing interventions have largely focused on technical modifications, such as instructing bowlers to maintain concentric contractions of the hip and knee extensors upon front foot contact. These modifications are often accompanied by adjustments in stride length and plant angle to position the front leg further from vertical at impact [13]. However, altering front leg configuration is challenging, as ground reaction forces during early front foot contact are extremely high [7,13].

A coaching intervention within the England and Wales Cricket Board (ECB) elite fast-bowling programme, for example, failed to modify knee angles significantly despite expert instruction [26]. Similarly, in younger medium-fast bowlers (16.6 ± 0.7 years), peak concentric knee extension torque was not significantly correlated with knee kinematics at front foot contact or ball release [12]. Collectively, these findings suggest that the front leg technique may be resistant to change through strength or cue-based training alone.

To support more effective coaching interventions, researchers should examine the causal mechanisms governing front leg motion from front foot contact to ball release. Kinetic analysis is therefore a primary tool for determining how joint torques actuate front leg segments [14]. Middleton et al. [14] calculated peak hip and knee powers in 30 male fast bowlers and found that only positive hip power differentiated higher-performing from amateur bowlers. Although time-variant data were not published, they described a support phase followed by a drive phase inferred from the transition between negative and positive hip and knee powers. Ferdinands et al. [27,28] applied a similar approach to lumbar spine and rear leg mechanics, categorising torque–motion patterns as controlled, active, stabilising, or negligible, based on joint power (the product of torque and angular velocity). Such analyses reveal the magnitude and type of net muscle actions that govern joint motion. Applying similar approaches to the front leg could clarify the mechanical functions that contribute to different front leg techniques.

Another approach to examining muscle control within a joint is to calculate the angular impulse, which accounts for the cumulative impact of torque over a specific duration [29]. Stefanyshyn et al. [30] used this method to quantify knee angular impulse during the stance phase of running, providing insight into how cumulative torque influences patellofemoral loading. Joint power and angular impulse analyses therefore offer complementary insights: power characterises the instantaneous mechanical role of a joint (concentric vs. eccentric), whereas angular impulse reflects the time-integrated torque responsible for changes in joint angle. Applying both metrics to the front leg enables a deeper understanding of the mechanisms that produce flexor, flexor–extender, extender, or braced techniques.

Cricket coaching faces a specific challenge: although evidence supporting the mechanical benefits of an extended front knee is limited, many coaching frameworks still promote transitions from flexor to extender or braced techniques with extended knees. This ongoing focus highlights the need for evidence-based biomechanical analysis. Studying the kinetics of front leg control and activation strategies in fast bowlers could offer valuable insights for refining technical models and enhancing the mechanical efficiency of the delivery stride.

It is also important to determine whether front leg kinetics and anthropometric characteristics make independent contributions to ball release speed, beyond descriptive technical patterns. Clarifying these relationships can help establish whether commonly coached cues, such as promoting a more extended front knee, retain predictive value once confounding factors such as run-up speed are controlled. Therefore, the aims of this study were the following: (1) to examine the dynamic control and activation of the front hip and knee using joint power and angular impulse analyses, and (2) to determine the extent to which front leg kinematics and anthropometric factors predict ball release speed under multivariate conditions.

## 2. Materials and Methods

### 2.1. The Sample

The sample consisted of eighteen young fast bowlers (mean age = 17.2 ± 1.7 years; mean height = 1.88 ± 0.05 m; mean mass = 86.0 ± 10.0 kg). All participants were actively engaged in regional or New South Wales (Australia) state-level cricket training programmes and had no history of injury in the previous six months. Ethical approval for this study was obtained from the Human Research Ethics Committee of the University of Sydney (approval nos. 10266; 2 March 2010, and 2012/1916; 30 April 2015). Written informed consent was obtained from all participants.

### 2.2. Data Collection

All trials were performed in a biomechanics laboratory, in which bowlers could achieve a full-length run-up on a Mondo athletics track. A 14-camera Cortex Motion Analysis System (Version 1.0, Motion Analysis Corporation, Rohnert Park, CA, USA) was employed as the primary kinematic sensing system, capturing three-dimensional (3D) motion at 200 Hz [31]. Each camera functioned as an optical sensor equipped with infrared light-emitting diodes (LEDs) and high-speed image sensors to detect reflective markers [32] attached to the athlete’s body and the cricket ball. The system triangulated marker positions across cameras to yield precise 3D spatial coordinates, allowing for the detailed reconstruction of body segments and joint motion [33].

Ground reaction forces were recorded at 1000 Hz using two Kistler 9287BA force plates (Kistler, Winterthur, Switzerland) embedded flush with the laboratory floor. These plates serve as multi-axis piezoelectric force sensors, measuring three orthogonal components of force and corresponding moments during foot contact [34]. The synchronised force and motion capture data streams provided input for the subsequent calculations of joint angular kinematics and kinetics.

Each participant performed 6 bowling trials. All trials were conducted in a biomechanics laboratory with a full-length run-up on a Mondo athletics track, enabling participants to perform deliveries under realistic conditions. Bowling trials were deemed successful when the back and front feet contacted the first and second force plates, respectively, during the delivery stride. If either foot did not make full contact on the corresponding force platform, then that trial was discarded and subsequently replaced with the next successful trial. Before the bowling trials, a standard static trial was used for each bowler to establish a reference anatomical position and calculate joint angles [35].

Before the formal bowling trials, each bowler was asked to warm up under match conditions and permitted to bowl practice deliveries while wearing the full marker set to become familiarised with the laboratory testing conditions. Testing began when the bowler declared himself warmed up and ready to bowl. Each participant was then instructed to bowl at maximum effort and pitch the ball within a “good length” area demarcated by two white lines, 13 m and 19 m from the stumps at the bowler’s end. The capture volume was sufficient to permit the recording of the entire bowling action, including the times of back foot contact, front foot contact, and ball release, as well as the follow-through phase, which ended when the bowling arm was oriented vertically downwards. The trial with the fastest ball speed was selected for analysis. The Cortex Motion Analysis System was calibrated according to the manufacturer’s recommendations, resulting in a residual error of less than 1 mm in marker position.

### 2.3. Marker Configuration and Motion Capture Protocol

For motion analysis capture, each participant wore a full-body marker set comprising fifty-one 25 mm spherical markers, which were attached to bony landmarks [27]. Markers were located on the left and right sides of the body, except for markers halfway between the posterior superior iliac spines (mid-PSIS), seventh cervical vertebrae, supra-sternal notch, and head. Body segment inertial parameters were scaled to each participant using de Leva’s [36] adjustments to the Zatsiorsky–Seluyanov model. Segment masses, radii of gyration, and centre of mass locations were not applied as fixed constants; instead, they were normalised to each bowler’s measured body mass and segment lengths derived from the 51-marker full-body model, ensuring subject-specific inertial parameter estimates appropriate for adolescent athletes. Exceptions to this practice were markers placed on the shoulders, hips, and cricket ball. There were three markers placed on the anterior superior iliac spine, mid-PSIS, and greater trochanter, according to the marker placements recommended by Bell, Pederson, and Brand [37], to calculate the hip joint centres. All other joint centres were calculated as the average position between two markers placed either medially and laterally or anteriorly and posteriorly on the joint.

To calculate ball kinematics, three markers were attached to the surface of the cricket ball, according to the protocol used in Beach et al. [38,39]. Ball release was defined as the first frame when the ball separated from the hand, a distance that was determined by both the ball’s COM velocity and acceleration data, as well as a visual inspection of the raw motion analysis data of the bowling hand.

A recursive fourth-order low-pass Butterworth filter was used to smooth the *x*-*y*-*z* coordinates of the marker data. The choice of suitable cut-off frequencies was determined by residual analysis [40], a process that requires some subjective assessment to fine-tune the values [35]. The corresponding acceleration–time curves were also observed. Through these processes, a cut-off frequency of 18 Hz was selected.

### 2.4. Inverse Dynamic Model of Front Leg

The smoothed motion analysis data of the markers were imported into a four-segment rigid-body model of the front leg designed using Visual3D software (Version 5, C-Motion, Germantown, MD, USA). The local segment coordinate systems of the rigid-body model were defined for the pelvis and front leg, based on the methodology of Grood and Suntay [41]: the adduction–abduction axis calculated as the cross-product of the long-axis vector and the vector from a distal lateral joint marker to the proximal joint centre; and the flexion–extension axis calculated as the cross-product between the long-axis vector and the adduction–abduction axis vector.

Joint angles were derived using the joint coordinate system approach, with angles calculated from the distal segment relative to the proximal segment and the pelvis established as the root segment [42]. Hip and knee extension–flexion angular impulses were calculated about the proximal ends of the thigh and shank segments (i.e., calculated about the hip and knee joints, respectively) and normalised over the period from front foot contact (0%) to ball release (100%), a period that approximately corresponds to the arm-acceleration phase (or power phase) in bowling [6,27,28].

### 2.5. Joint Power Analysis

Joint power, calculated as the product of joint torque and angular velocity, was used to characterise front leg motion as either active, controlled, or negligible, based on a modification of the protocol established by Winter and Sienko [43] and Ferdinands et al. [27], where joint torque was normalised over body mass × height. Both joint power and angular impulse calculations were used to quantify the control patterns of front leg motion.

Active motion was defined when the joint power was positive, corresponding to power generation or net concentric muscle action. Controlled motion was defined when joint power was negative, corresponding to power absorption or net eccentric muscle action. Joint power was considered negligible when the absolute value of the average joint power over a period was less than the baseline power value of 5.0 W kg^−1^ m^−1^. Extension motion was defined when angular velocity was positive. Static motion was defined over any period when the change in knee angle was less than 10°.

### 2.6. Joint Angular Impulse Analysis

Joint angular impulses were generated by calculating the areas under the torque curves that corresponded to discrete periods of knee extension and flexion in the joint angular kinematic data. Since extension was defined as positive, a positive area produces a positive joint angular impulse.

To classify the knee action types, we calculated the angular impulse effect on knee joint angle, which is a modification of the joint torque motion-effect concept that was applied to rear leg motion in fast bowlers by Ferdinands, Sinclair, Stuelcken, and Green (Table 1) [28]. The percentile ranks (P) of the absolute values of the normalised knee joint angular impulse from the sample were used to relatively quantify the strength of the joint torque effect: P ≤ 25, negligible; 25 < P ≤ 50, moderate; P > 50, strong. When a normalised joint angular impulse greater than 25 P acted in the same direction as the change in joint angle, then the corresponding effect was defined as active. Conversely, when a normalised joint angular impulse greater than 25 P acted in the opposite direction to the change in joint angle, then the corresponding torque effect was defined as controlled. In contrast, if a normalised joint angular impulse was less than or equal to 25 P, then the torque effect was determined to be negligible, and the magnitude and/or direction of the change in joint angle does not matter.

### 2.7. Front Limb Action Classification

The kinematic classification of front knee motion over the arm-acceleration phase (defined from front foot contact to ball release) was based on modifying the threshold criteria used by Portus, Mason, Elliott, Pfitzner, and Done [7] and Beach, Ferdinands, and Sinclair [39]. Knee action mechanics were categorised into three primary techniques, which were subsequently subdivided into nine secondary techniques during the arm-acceleration phase (Table 2). The secondary techniques are characterised by identifying intervals of change and/or stability in the knee flexion–extension angle spanning from 0% to 100% of the arm-acceleration phase. Another classification, termed the front leg lever type, was established based on the knee angle at ball release, where a knee angle of 180º indicates a fully extended knee:

Extended Knee (EK): Front knee angle ≥ 170°.

Flexed Knee (FK): Front knee angle < 170°.

To illustrate this, consider the following example. Classify a fast bowler who undergoes distinct changes in knee angle across three specific segments of the arm-acceleration phase, as shown below:

1. The 10–15% arm-acceleration phase: Knee angle transitions from 158° to 169° (classified as extender).
2. The 16–55% arm-acceleration phase: Knee angle shifts from 169° to 148°, denoting a 21° increase in knee flexion (classified as flexor).
3. The 56–100% arm-acceleration phase: Knee angle changes from 148° to 160°, indicating a 12° increase in knee extension (classified as extender).

Evaluation: The bowler is classified as an (extender)–flexor–extender, with a front leg lever type of flexed knee since the knee flexion–extension angle is below 170° at ball release. Note that the formal classification of knee action specifically considers the last two periods of the arm-acceleration phase. The term “extender” in parentheses is included for completeness, but the primary classification remains as flexor–extender.

### 2.8. Data Analysis

The trial with the highest ball speed, which satisfied the accuracy constraint of the ball landing within the area demarcated by two white lines, 13 m and 19 m from the stumps at the bowler’s end, was selected for analysis on the basis that it represented peak performance.

All statistical analyses of peak performance trials were conducted in MATLAB R2023b (MathWorks, Natick, MA, USA). A multiple linear regression model was then developed to evaluate the extent to which front leg kinematics and anthropometric variables predicted ball release speed. Ball speed (m·s^−1^) served as the dependent variable, and four predictors were entered based on established biomechanical relevance: knee angle at front foot contact (°), run-up speed at back foot contact (RunUpSpeed_BFC; m·s^−1^), standing height (cm), and body mass (kg). The run-up speed was taken as the component of the centre of mass velocity in the direction of the wicket line at back foot contact.

For each predictor, unstandardised coefficients (β), standard errors, t-statistics, *p*-values, and standardised beta coefficients (β*) were extracted. Overall model performance was assessed using the coefficient of determination (R^2^), adjusted R^2^, standard error (SE), and the model F-statistic.

Regression assumptions were evaluated systematically [44] (Table 3). Residual normality was assessed using the Shapiro–Wilk test and inspection of Q–Q plots [45]. Homoscedasticity was examined using the Breusch–Pagan test [46]. Multicollinearity was evaluated using variance inflation factors (VIFs), with values < 5 considered acceptable [47]. Influence diagnostics were evaluated using Cook’s distance (threshold = 4/*n*) to identify potential influential observations [48].

For any significant predictor in the model, a partial regression (added-variable) plot was generated to visualise its unique contribution to ball speed after adjusting for the covariates. The fitted line represents the adjusted linear relationship between the predictor and outcome.

To illustrate the movement patterns of each technique classification, ensemble averages were calculated and graphed with envelopes of one standard deviation for the hip and knee joint powers of each participant within each knee-action type [27,49].

## 3. Results

Model assumptions were satisfied (Table 3). The residuals were normally distributed (Shapiro–Wilk *p* = 0.317), multicollinearity was low (all VIF < 1.5), and homoscedasticity was acceptable (Breusch–Pagan *p* = 0.081). Influence diagnostics identified one participant (Sub5) with an elevated Cook’s distance (D = 0.485), though within acceptable bounds (threshold = 4/*n* = 0.222). Retaining this observation did not alter model conclusions, and the result remained stable.

Table 4 presents the descriptive statistics for all variables included in the regression analysis.

The multiple regression model explained 47.2% of the variance in ball release speed (R^2^ = 0.472; adjusted R^2^ = 0.31), representing a moderate predictive effect. The overall model approached statistical significance (F(4,13) = 2.91; *p* = 0.064) (Table 5).

**Figure 1:**
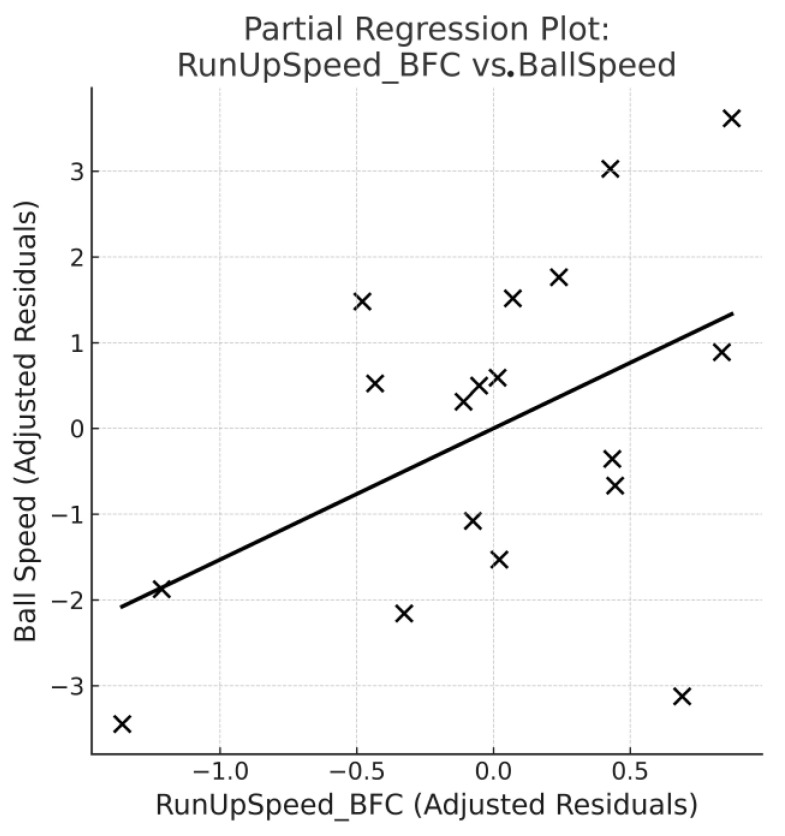
A partial regression plot showing the unique association between run-up speed at back foot contact (RunUpSpeed_BFC) and ball release speed after adjusting for knee angle, height, and mass. Points represent individual bowlers (*n* = 18). The line indicates the adjusted linear relationship.

Table 5 shows that RunUpSpeed_BFC emerged as the strongest predictor of ball speed (β = 1.53 ± 0.78 m·s^−1^; *p* = 0.071), indicating a meaningful trend whereby faster approach velocity contributed to higher ball velocity. The standardised beta for RunUpSpeed_BFC (β* = 0.45) further confirmed its relative importance compared with other predictors. Because run-up speed was the only predictor approaching significance in the multivariate model, a partial regression plot was created to visualise its independent contribution (Figure 1). This plot shows the adjusted residuals of run-up speed plotted against the adjusted residuals of ball speed after accounting for height, mass, and knee angle. The upward trend confirms that a faster approach velocity is associated with higher ball speed beyond the effects of other variables.

Knee angle at front foot contact demonstrated only a small and non-significant effect on ball speed (β = 0.025 ± 0.018; *p* = 0.20). Practically, a 10° increase in knee extension corresponded to an estimated 0.25 m·s^−1^ increase in ball speed, which is less than 1% of the group mean, indicating a negligible performance impact.

Height (*p* = 0.34) and mass (*p* = 0.10) did not contribute significantly to the model after accounting for RunUpSpeed_BFC.

**Figure 2:**
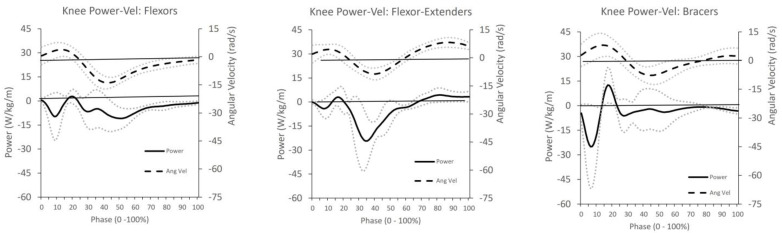
The ensemble average ± SD of knee flexion–extension velocity and muscle power for the flexor, flexor–extender, and extender–bracer groups from front foot contact to ball release. (Power, solid black line; angular velocity, dashed line; ± SD, dotted lines; zero reference lines, dashpot.)

**Figure 3:**
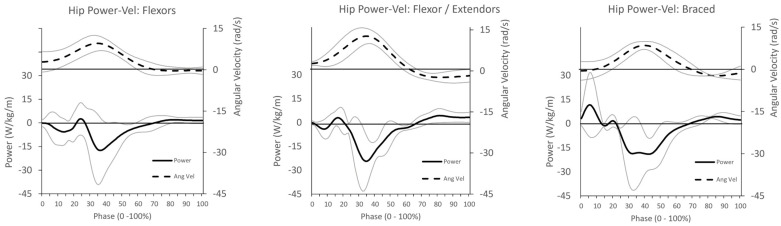
The ensemble average ± SD of hip flexion–extension velocity and muscle power for the flexor, flexor–extender, and extender–bracer groups from front foot contact to ball release. (Power, black line; angular velocity, dashed line; ±SD, grey lines; zero reference lines, dashpot.)

Joint angular velocity and normalised muscle power about the knee joint shown were similar for all groups (Figure 2; Table 6). However, joint angular velocity and normalised muscle power about the hip joint displayed different time-variant characteristics for these groups (Figure 3; Table 7). The bowlers were classified under the following front leg lever types: 10 flexors, 5 flexor–extenders, and 3 extenders (Table 8).

For the 18 bowlers, Table 8 shows the change in knee angle, knee angle at ball release, normalised knee joint angular impulse, and percentile rank of normalised knee joint angular impulse for each discrete period of knee flexion and extension from front foot contact to ball release, leading to the determination of front knee action type, front leg lever type, and angular impulse motion effect.

## 4. Discussion

Biomechanics and the coaching literature generally recommend that fast bowlers develop a front knee that is extended at the time of ball release. Accordingly, the front leg techniques typically promoted are the flexor–extender, extender, and bracer techniques, whereas the flexor technique is not advocated. Therefore, this study aimed to determine whether (i) the mechanical processes underpinning the recommended techniques differ from those of the flexor technique and (ii) a more extended front knee at ball release is associated with faster ball speeds.

### 4.1. Kinematic Analysis

This study examined whether front leg kinematics and selected anthropometric variables predicted ball release speed in junior fast bowlers. The multiple regression model explained approximately 47% of the variance in ball speed, with run-up speed at back foot contact (RunUpSpeed_BFC) emerging as the strongest predictor. This finding is consistent with previous work demonstrating that a faster approach velocity enables greater whole-body momentum entering the delivery stride [12,13], thereby facilitating more effective momentum transfer through the kinetic chain and contributing meaningfully to ball release speed. The trend-level effect of RunUpSpeed_BFC in this cohort highlights its importance even among relatively young and developing bowlers.

In contrast, knee angle at front foot contact demonstrated only a small and non-significant contribution to ball speed. The unstandardised coefficient (β = 0.025 m·s^−1^·deg^−1^) indicates that even a 10° change in knee configuration would alter ball speed by only ~0.25 m·s^−1^, representing a trivial practical influence. Although the standardised beta for KneeAngle appeared moderate in magnitude, this reflects the large natural variability in knee angle values rather than a meaningful causal effect. When interpreted alongside the non-significant *p*-value (*p* = 0.20), it becomes clear that front knee position at front foot contact is not an independent predictor of ball speed after accounting for run-up speed.

These findings are consistent with previous research reporting weak or inconsistent associations between knee kinematics and ball release speed [12,13,14]. Worthington et al. [13] found that knee angle explained just 13.4% of ball speed variance after controlling for run-up velocity, while Middleton et al. [14] found no significant differences in knee angles between elite and amateur bowlers. This pattern suggests that earlier bivariate correlations may have been influenced by confounding factors: positively by the preferential selection and coaching of bowlers who naturally exhibit a more extended front leg or negatively by substantial inter-individual differences in bowling mechanics—rather than reflecting a direct causal effect of knee extension on ball speed.

Model diagnostics indicated that statistical assumptions were satisfied (Shapiro–Wilk *p* = 0.317; Breusch–Pagan *p* = 0.081; all VIFs < 1.5). One participant (Sub5) demonstrated a moderately elevated Cook’s distance, reflecting an unusual combination of high run-up speed and substantial knee flexion. However, retaining this observation did not meaningfully alter model estimates or significance values, supporting the stability and robustness of the findings.

Overall, the results highlight the multifactorial nature of ball speed generation in fast bowling and suggest that factors related to whole-body momentum generation, particularly run-up speed, are more influential than isolated front leg kinematic parameters. Technical interventions may therefore benefit more from optimising approach velocity, proximal sequencing, and whole-body coordination rather than emphasising front knee extension at front foot contact as a stand-alone performance determinant.

In the present study, most bowlers (61.1%; *n* = 10) were classified as flexors, indicating that the major phase of knee motion occurred during the latter portion of the arm-acceleration phase. Consistent with previous research [7,14], a smaller proportion of bowlers (28%; *n* = 5) demonstrated the extender class of techniques, all of which were flexor–extender in nature. Two bowlers exhibited bracer techniques, comprising two extender–bracers and one flexor–bracer. Overall, 33% (*n* = 6) of the bowlers achieved a straight front leg lever at ball release. Despite the predominance of flexor techniques in this cohort, much of the existing coaching literature continues to advocate for bracer or extender front leg actions as the mechanically optimal configuration [22,24]. These results highlight a potential discrepancy between commonly promoted coaching ideals and the movement strategies most naturally adopted by developing fast bowlers, suggesting that the front leg technique may reflect individual biomechanical preferences or coordination strategies rather than a single universally optimal pattern.

It is acknowledged that research attempting to establish a relationship between front knee angle at ball release and ball speed is inherently challenging due to numerous sources of variability. Inter-subject differences in anthropometry, strength, coordination, and preferred technique, as well as variability in effort and testing conditions on the day, introduce substantial noise into the data. Measurement limitations, including marker placement error, camera resolution, and inconsistencies in kinematic capture, further constrain the reliability of the findings. While intra-subject designs could theoretically control for some of these factors, they are practically difficult, as many deliveries would be required at consistent maximal effort, which is challenging to achieve and verify. Self-reported effort is unlikely to provide the resolution needed to confirm consistent ball velocity across repetitions. Moreover, only small variations in the knee angle are likely to occur naturally, and it remains uncertain whether any systematic relationship between front knee configuration and ball speed exists.

Interpreting kinematic research on the front leg technique is further complicated by pre-selection effects in coaching pathways. Many elite programmes prioritise bowlers with braced front leg techniques, giving them greater access to high-level coaching, practice facilities, and performance monitoring. Bowlers naturally using flexor techniques may therefore have reduced opportunities to train in professional environments, while coaches may also lack expertise in teaching flexor-based mechanics effectively. This may create a potential cognitive bias affecting the available data [50,51], favouring the extender technique, as observed differences may reflect selection and opportunity rather than inherent biomechanical superiority [52]. Hence, we performed power and angular impulse analyses to widen the perspective of the mechanisms that underlie the front leg technique.

### 4.2. Power Analysis

While coaches frequently encourage the extended front knee technique, its practical implementation appears challenging. Most bowlers naturally employ a flexor technique, and modifying established movement patterns can be difficult. Addressing this issue requires detailed biomechanical analysis to understand the interaction between joint power and coordination, shedding light on the intricacies of technique transitions and informing targeted coaching strategies.

Muscle power analysis provides valuable insight into the nature of torque actuation at a joint, revealing how force and motion interact to generate effective movement patterns [27]. In the context of fast bowling, such analysis can help identify and correct excessive front knee flexion often observed in bowlers employing a flexor technique. In the present study, seven bowlers (38.9%) demonstrated a knee angle of less than 130° at ball release, indicating more than 50° of flexion, which may exceed the range required for efficient energy transfer through the kinetic chain. While moderate knee flexion can facilitate impact absorption, excessive flexion may reduce the mechanical coupling of the front leg with proximal segments, thereby constraining the transfer of momentum from the trunk and pelvis to the bowling arm. Calculating power at the front hip and knee enables an evaluation of these mechanical distinctions between flexor techniques with differing amounts of knee flexion. A key question is whether bowlers exhibiting excessive flexion can improve efficiency by generating greater knee extension power, effectively transitioning toward a more functional flexor or flexor–extender pattern. If front leg extension is primarily achieved through active (concentric) muscle action, then targeted strength interventions focusing on the knee extensors may be beneficial. Conversely, if extension arises mainly from controlled (eccentric) or momentum-driven interactions between segments, then coaching interventions emphasising coordination and timing rather than isolated joint strengthening may be more effective.

This study found that controlled power dominated during the arm-acceleration phase, particularly among bowlers using the flexor technique, who relied on the eccentric control of both hip and knee flexors. Following an initial period of knee extension (0–25%), these bowlers primarily displayed controlled power across the remainder of the phase (Figure 2; Table 6). The flexor–extender group also exhibited predominantly controlled knee motion, especially during the 65–100% phase of knee extension, with minimal power evident. These observations suggest that both front knee extension and flexion often occur without active torque generation at the knee joint, indicating that external dynamic factors, such as segmental interactions or velocity-dependent torques [53], may play a more significant role than local muscle activation. Hence, the process of regulating “excessive” knee flexors is unlikely to succeed with interventions that focus locally on reducing knee angle.

In the knee bracer group, controlled power was again prevalent (Figure 2; Table 6). Initially, all three bowlers extended their knees under controlled power, but once the knee stabilised, angular velocity no longer corresponded with power development, suggesting minimal local torque. This pattern also indicates that the braced position likely results from segmental dynamics elsewhere in the kinetic chain rather than direct knee extensor contraction.

Across all groups, hip flexors primarily exhibited controlled power, especially during peak hip flexion (Figure 3; Table 7). This finding indicates that hip motion, like knee motion, is influenced by non-local dynamics within the kinetic chain. Consequently, coaching interventions that focus solely on hip extension to reduce excessive knee flexion or promote knee extension may be ineffective. Trunk and pelvic rotation are closely coupled with hip kinematics in rotational sport actions, and pelvis rotation and hip joint kinetics can contribute substantially to hip-related motion rather than relying solely on local concentric hip extensor contraction [54]. For instance, Wang et al. [55] reported that pelvic control in the axial plane, with the gluteus medius playing a key role, is strongly associated with pitching velocity, implying that proximal pelvic control and coordinated pelvis–trunk motion can drive hip mechanics, including hip extension components, in throwing actions. These findings suggest that hip extension in fast bowling is an emergent property of coordinated whole-body motion rather than the product of isolated muscular activation. These interdependencies highlight the complexity of movement coordination and the importance of a systems-based approach to technical training. This systems perspective implies that a well-designed training programme accounts for the predominance of controlled (eccentric) power patterns observed at the hip and knee. In fast bowling, front leg ground reaction forces can range between 4.0 and 8.6 bodyweights [56], placing substantial demand on the knee extensors and lumbopelvic stabilisers. These loads could be managed through controlled eccentric knee flexion; in contrast, a stiff, extended, “zero-response” landing would increase impact transmission and elevate anterior knee and lumbar stress [57].

Targeted exercises that strengthen eccentric knee flexion control, such as unilateral drop-landing or drop-jump progressions, have been shown to enhance lower limb stiffness regulation and neuromuscular coordination [58,59]. Similarly, controlled landing and deceleration training has been demonstrated to reduce knee joint loading and improve energy absorption strategies [57,60]. These approaches parallel the biomechanical demands of the bowling action and offer practical avenues for developing the skills of bowlers who can tolerate high GRFs while maintaining efficient momentum transfer.

Collectively, these insights demonstrate translational relevance in practice. Interventions that strengthen eccentric control and optimise whole-body coordination are likely to benefit both performance outcomes and injury resilience in junior fast bowlers, providing an actionable pathway for coaches and practitioners.

### 4.3. Angular Impulse Motion Effects

Angular impulse represents the change in angular momentum produced by torque over time [29]. An active angular impulse effect occurs when local torque acts in the same direction as joint motion, while a controlled effect occurs when torque opposes it (Table 1). In this study, most front knee extension–flexion cycles exhibited controlled impulse motion effects, with only one active period observed (Table 8). This result is consistent with the joint power analysis, which shows that hip and knee joint torques predominantly functioned to regulate or resist motion rather than initiate it.

With respect to the knee joint specifically, approximately 90% of knee extension periods exhibited moderate or strong control, reflecting the inhibitory action of knee flexors on extension (Table 8). These results are consistent with previous analyses of power and torque–motion interactions in the lumbar spine and rear leg [27,28], which similarly revealed predominant controlled or negligible effects, suggesting the influence of non-local segmental dynamics on joint motion.

The controlled impulse motion effect can be conceptually linked to remote motion induction, where motion arises from intersegmental dynamics. Conversely, an active impulse motion effect is more likely—though not necessary—to arise from motion, driven by local joint torque. These concepts align with the instantaneous and cumulative effects described by Hirashima et al. [53], who used induced-acceleration analysis to evaluate multi-joint coordination. Although the impulse motion framework does not possess the same mathematical formalism, its conceptual foundation is analogous. Either way, in multisegment systems, joint motion can be influenced by forces originating remotely within the kinetic chain [53,61,62,63], implying that the net joint moment does not fully determine angular momentum changes at that joint. Hence, it is more appropriate to conceptualise bowling as an integrated mechanical system in which motion at one joint can be induced by dynamic interactions elsewhere.

The contrast between the flexor and extender groups further illustrates this principle: the former relies on the eccentric control of knee extensors to moderate flexion, whereas the latter depends on the eccentric control of knee flexors to regulate extension (Table 8). The latter may at first seem counterintuitive, but bowlers employing an extender technique are unlikely to rely solely on concentric quadriceps contraction throughout the delivery stride. As the knee approaches full extension, eccentric activity from the knee flexor musculature (and non-local influences) can act to decelerate motion, regulating the rate of extension and reducing the risk of hyperextension or joint overload. This controlled braking function enables the knee to maintain stiffness and stability at ball release while protecting surrounding soft tissues from excessive strain. This could partially explain why bowlers using extender techniques exhibited a greater reliance on knee flexors than those using flexor techniques. It also suggests that knee extension is driven non-locally, originating from interactions elsewhere in the kinetic chain. It is necessary, therefore, to account for the potential cascading effects of adjustments elsewhere in the system when designing interventions.

Several theoretical arguments have been proposed to justify bowling with an extended knee at ball release, such as increasing the effective lever length to elevate hand speed and release height [4], promoting the rapid deceleration of the centre of mass to facilitate upper body acceleration analogous to a pole-vaulter’s plant [5], and enhancing energy transfer through the kinetic chain while a flexing knee may dissipate energy [25]. However, both the power and angular impulse analyses suggest that fast bowling is a highly integrated action in which local joint motion is influenced by non-local, cumulative effects across multiple segments. Consequently, these qualitative models are inherently simplistic and overlook the critical role of kinetics, system dynamics, and insights from neuroscience and motor control in developing a comprehensive and evidence-based theory of technical optimality.

Although forward simulation studies, such as that by Felton et al. [17], provide valuable mechanistic insight by identifying how a particular model and objective function can produce high ball speeds, any claim that a straight front leg is the universal “optimal” configuration must be qualified. Simulation optima are conditional on the model structure, the cost function, constraints (planar dynamics, torque generator formulations), and the single subject used to parameterise the model. Altering these elements by including injury risk penalties, neuromuscular constraints, dynamic systems theory, complexity, or three-dimensional rotational torques can produce different optima (i.e., different “best” solutions) [17,64,65]. A case in point is that of Mike Procter and other fast bowlers described as delivering “off the wrong foot,” a term that is somewhat misleading because it actually refers to the completion of the majority of the arm-acceleration phase prior to front foot contact [66]. This type of action cannot be adequately explained by current forward simulation models, which primarily generate the optimal solution in terms of front foot contact with an extended knee.

In addition, future forward simulation models should incorporate the critical contributions of three-dimensional rotational torques that are known to influence fast bowling performance. Key mechanisms such as shoulder–hip separation, trunk rotation, and the coordinated transfer of rotational energy to the bowling arm have been consistently identified as determinants of release velocity in cricket fast bowling, mechanisms that have also been observed in related athletic motions such as javelin throwing and baseball pitching [6,7,67,68,69]. A planar torque limitation restricts the generality of conclusions regarding any claims of technical optimality, including front leg configuration, because three-dimensional rotational dynamics are likely key determinants of energy transfer and ball release velocity [6]. Such a limitation may also lead to misleading conclusions by suggesting, for example, that a front-on action is inherently optimal and a side-on action inefficient, and by discouraging the latter on the basis of greater transverse rotation and lateral bending, despite these being fundamental components of fast bowling. It is essential to account for the role of three-dimensional rotational dynamics in energy transfer and ball release velocity in all types of bowling actions [6,69]. Notable examples of exceptionally fast bowlers who employed side-on actions include Jeff Thomson, widely regarded as one of the fastest bowlers in history [70]; Shoaib Akhtar; Shaun Tait; Lasith Malinga; Fidel Edwards; and several others, who consistently bowled at speeds exceeding 150 km/h with top speeds approaching or in excess of 160 km/h [71].

Mechanically, a flexed-but-stiff front leg could theoretically perform the same functional role as a straight leg during the critical pre-stretch/loading phase by enabling the controlled eccentric loading of muscle–tendon units (stretch–shortening cycle benefits) with the advantage of attenuating high impact forces [72,73]. Consequently, bowlers adopting flexor–bracer (Bowler 13) or flexor–extender techniques with flexed knees at ball release (Bowler 18) may achieve similar functional front leg stiffnesses to bowlers with extended knees (Table 8). It is worth noting that in throwing and striking sports, such as baseball, pitchers frequently use a flexed lead leg while achieving high ball velocities through timed pelvis–trunk rotation and hip–shoulder separation. In tennis and discus, athletes can achieve maximal distal speeds even during transient flight phases when their feet are not in contact with the ground, demonstrating that continuous foot contact is not strictly required after the pre-loading phase. Hence, in practical terms, once the musculature of the trunk and shoulder has been effectively pre-stretched and energy has been loaded into the system, subsequent changes in front leg configuration (even collapse or loss of ground contact) do not necessarily diminish the application of Newton’s third law. In the case of Bowler 9 (Table 5), the front knee was flexed more than 50°. However, before considering any intervention, it would be prudent to quantify the bowler’s performance level and determine exactly when in the arm-acceleration phase the front knee flexes at the highest rate. Such an approach qualifies as an individualised, constraint-led coaching process in contrast to a universal application of the straight leg technique. In addition, interventions that prescribe isolated exercises targeting the quadriceps or knee extensors to alter knee mechanics without considering the intricate coordination of whole-body mechanics are difficult to justify.

The modest sample size (*n* = 18) reflects practical constraints common in high-resolution motion analysis research, where laboratory-based marker tracking, force platforms, and inverse dynamic modelling place logistical limits on participant testing. Although the multiple regression model in this study accounted for 47% of the variance in ball speed, the small sample size inherently reduces statistical power and increases uncertainty around effect estimates, particularly for smaller predictors such as knee angle. Inter-subject variability in anthropometry, run-up strategies, and coordination patterns adds further noise that may obscure true but subtle associations. Similar challenges occur in other data-sparse scientific domains (e.g., rare disease modelling), where methodological rigour, controlled protocols, and balanced data handling have been shown to improve inference despite limited samples [74]. In the present study, standardised testing procedures, a single best-performance trial, and multivariate modelling were used to counter, but not eliminate, this sample-related limitation. As such, the findings should be interpreted cautiously and viewed as preliminary evidence requiring confirmation in larger cohorts.

The use of each bowler’s fastest valid delivery was intended to ensure the analysis of peak performance mechanics. However, this may overrepresent the maximal rather than the typical technique. It is acknowledged that this approach prioritises performance-oriented interpretation but may not fully characterise habitual movement variability.

The percentile thresholds (≤25%, 25–50%, >50%) used to classify angular impulse magnitudes were adopted to maintain consistency with prior analyses of joint motion effects in fast bowling. However, these thresholds have not been empirically validated and should therefore be interpreted as heuristic boundaries rather than biomechanically grounded cut-offs. Future work is needed to evaluate the stability of these categories through quantitative approaches such as sensitivity analyses and cross-validation against reference kinetic patterns. Continuous modelling approaches may also provide a more robust alternative to discrete classification in larger datasets. Despite this limitation, the present findings provide a meaningful contribution to the emerging understanding of front leg mechanics in fast bowling, particularly in relation to technical optimisation and potential injury pathways.

## 5. Conclusions

This study examined front leg mechanics in junior fast bowlers using three-dimensional motion analysis, joint power and angular impulse evaluation, and a multivariate regression framework. The muscle power and impulse analyses showed that bowlers predominantly adopted flexor-based front leg strategies, with controlled (eccentric) power patterns evident at both the hip and knee. These findings challenge traditional assumptions that a rigid, “braced” front leg represents an inherently superior technical model and instead support the interpretation that front leg behaviour emerges from coordinated, whole-body dynamics across the kinetic chain.

The inclusion of multiple linear regression strengthened these observations by quantifying the relative contribution of key variables to ball release speed. Run-up speed at back foot contact (RunUpSpeed_BFC) was the strongest predictor of ball speed, whereas knee angle at front foot contact demonstrated a small, non-significant, and practically trivial effect once other factors were controlled. These results indicate that knee configuration at front foot contact is not an independent determinant of performance and should not be prioritised in isolation within coaching interventions.

Overall, the biomechanical and statistical findings suggest that front leg motion should be understood as part of a systems-level coordination strategy rather than the outcome of isolated local joint actions. Future research should incorporate three-dimensional rotational dynamics, whole-body momentum transfer, and broader inter-joint coupling to explain performance variability. From an applied perspective, training interventions may be more effective when they focus on optimising whole-body coordination, eccentric control during the braking phase, and the timing of energy transfer, rather than attempting to modify front leg mechanics through isolated strengthening or technique drills.

## Figures and Tables

**Table 1:** Definition of angular impulse motion effects on knee joint based on normalised knee joint angular impulse and change in knee joint angle.

Angular Impulse Motion Effect	Normalised Joint Angular Impulse Threshold	Change in Joint Angle Criterion	Joint Angular Impulse vs. Joint Angle Sense
Active	|τ.∆t|>25 P	Θ_knee_ > 10°	Same direction
Controlled	|τ.∆t|>25 P	Θ_knee_ > 10°	Opposite direction
Stabilised	|τ.∆t|>25 P	Θ_knee_ ≤ 10°	Either direction
Negligible	|τ.∆t|≤25 P	Θ_knee_ ε ℛ (i.e., all real values	Either direction

**Table 2:** Classification of front knee action types into primary and secondary techniques for last two periods of change in knee joint angle prior to ball release during arm-acceleration phase.

Class	Technique	Final Two Periods of Change in Knee Joint Angle
Bracer	Constant Bracer Flexor–Bracer Extender–Bracer	Δ knee angle < 10°(i.e., flexion or extension) Δ knee flexion ≥ 10° followed by Δ knee angle < 10° Δ knee extension ≥ 10° followed by Δ knee angle < 10°
Flexor	Continuous Flexor Bracer–Flexor Extender–Flexor	Δ knee flexion ≥ 10° Δ knee angle < 10° followed by Δ knee flexion ≥ 10° Δ knee extension ≥ 10° followed by Δ knee flexion ≥ 10°
Extender	Continuous Extender Bracer–Extender Flexor–Extender	Δ knee extension ≥ 10° Δ knee angle < 10° followed by Δ knee extension ≥ 10° Δ knee flexion ≥ 10° followed by Δ knee extension ≥ 10°

**Table 3:** Regression diagnostics and assumption checks.

Diagnostic Test	Statistic	*p*-Value	Interpretation
Shapiro–Wilk (normality)	—	0.317	Normal residuals
Breusch–Pagan (homoscedasticity)	3.036	0.081	Acceptable
VIF—Knee Angle	1.178	—	Low collinearity
VIF—RunUpSpBFC	1.278	—	Low collinearity
VIF—Height	1.390	—	Low collinearity
VIF—Mass	1.401	—	Low collinearity
Max Cook’s Distance	0.485	—	Sub5 influential but acceptable

**Table 4:** Descriptive statistics of multiple regression variables.

Variable	Mean	SD	Min	Max
BallSpeed	33.0	2.3	29.4	38.4
KneeAngle	−25.7	20.5	−54.6	20.9
RunUpSpeed_BFC	5.8	0.7	4.3	6.88
Height	188.0	5.2	181.9	200.8
Mass	86.0	10.0	64.4	100.0

**Table 5:** Regression coefficients predicting ball speed.

Term	β	SE	t	*p*	Β*
Intercept	36.733	20.553	1.787	0.097	—
KneeAngle	0.0247	0.0183	1.349	0.2	0.30
RunUpSpeed_BFC	1.5298	0.7767	1.97	0.071	0.45
Height	−0.1111	0.1114	−0.997	0.337	–0.24
Mass	0.104	0.0596	1.745	0.105	0.42

**Table 6:** Knee action of three most common groups. Dashed lines indicate types of knee joint actuation.

	Flexor Group		Flexor–Extender Group		Knee Bracer Group	
Phase (%)	Knee Motion	Actuation	Knee Motion	Actuation	Knee Motion	Actuation
0	Extension	Controlled	Extension	Negligible	Extension	Controlled
5						
10						
15						
20					Extension	Active
25	Flexion	Controlled	Flexion	Controlled	Extension	Negligible
30						
35					Flexion	Negligible
40						
45						
50						
55						
60						
65						
70						
75			Extension	Negligible		
80					Static	
85						
90						
95						
100						

**Table 7:** Hip action of three most common classification groups.

	Flexor Group		Flexor–Extender Group		Knee Bracer Group	
Phase (%)	Hip Motion	Actuation	Hip Motion	Actuation	Hip Motion	Actuation
0	Flexion	Controlled	Flexion	Negligible	Flexion	Negligible
5						
10						
15						
20						
25	Flexion	Negligible	Flexion	Controlled	Flexion	Controlled
30	Flexion	Controlled				
35						
40						
45						
50						
55						
60						
65						
70			Static	Negligible	Static	Negligible
75	Static	Negligible				
80						
85						
90						
95						
100						

**Table 8:** Classification of front leg motion based on percentile ranks of normalised knee joint angular impulse data, arranged in terms of knee action type.

Sub	Knee Extension–Flexion Periods (% Phase)	Change in Knee Angle Extension + Flexion −	Knee Angle at Ball Release Extended Knee (EK) Flexed Knee (FK)	Knee Action Type	Normalised Knee Joint Angular Impulse (N·m·s·kg^−1^)	Percentile Rank (P)	Angular Impulse Motion Effect
1	0–100%	−45.8°	126.4° (FK)	Continuous Flexor	+20.9	57.5	Strong Controlled
5	0–100%	−54.6°	110.9° (FK)	Continuous Flexor	+54.4	80	Strong Controlled
6	0–29% 30–100%	<10.0° −27.2°	147.6° (FK)	Bracer Flexor	−8.0 +4.8	35.0 22.5	Mod. Stabiliser Negligible
7	0–22% 23–100%	<10.0° −27.2°	138.4° (FK)	Bracer Flexor	−12.4 +30.6	42.5 72.5	Mod. Stabiliser Strong Controlled
8	0–20% 21–100%	<10.0° −44.9°	121.8° (FK)	Bracer Flexor	+0.1 +80.0	2.5 95	Negligible Strong Controlled
9	0–19% 20–100%	<10.0° −37.4°	127.8° (FK)	Bracer Flexor	−5.5 +87.8	25 100	Negligible Strong Controlled
11	0–25% 26–100%	<10.0° −39.2°	125.7° (FK)	Bracer Flexor	−2.8 +69.7	12.5 87.5	Negligible Strong Controlled
14	0–20% 21–100%	<10.0° −43.6°	128.5° (FK)	Bracer Flexor	+3.8 +72.7	20.0 92.5	Negligible Strong Controlled
15	0–22% 23–100%	<10.0° −38.9°	129.8° (FK)	Bracer Flexor	−18.5 +64.8	55 85	Strong Stabiliser Strong Controlled
17	0–25% 26–100%	<10.0° −32.3°	135.1° (FK)	Bracer Flexor	−7.6 +69.9	27.5 90.0	Mod. Stabiliser Strong Controlled
18	0–33% 34–59% 60–100%	<10.0° −12.0° +10.5°	144.4° (FK)	(Bracer) Flexor Extender	+42.3 +49.6 +23.2	75.0 77.5 60.0	Strong Stabiliser Strong Controlled Strong Controlled
2	0–7% 8–58% 59– 100%	<10.0° −17.7° +19.7°	175.8° (EK)	- Flexor Extender	−0.9 +13.3 +7.8	7.5 45.0 32.5	Negligible Mod. Controlled Mod. Active
3	0–19% 20–56% 57–100%	<10.0° −12.5° +18.7°	178.8° (EK)	(Bracer) Flexor Extender	−0.6 +27.5 −17.2	5 62.5 52.5	Negligible Strong Controlled Strong Controlled
4	0–30% 31–66% 67–100%	+10.9° −15.5° +17.3°	175.0° (EK)	(Extender) Flexor Extender	+1.3 +28.2 −8.7	10 67.5 37.5	Negligible Strong Controlled Mod. Controlled
12	0–23% 24–59% 60–100%	+10.2° −16.2° +20.9°	190.0° (EK)	(Extender) Flexor (Hyper)Extender	−17.0 +3.0 −27.7	50 15 65	Strong Controlled Negligible Strong Controlled
10	0–27% 28–100%	+12.4° <10.0°	172.6° (EK)	Extender Bracer	−15.7 −7.6	47.5 27.5	Mod. Controlled Negligible
16	0–34% 35–100%	+18.6° <10.0°	181.8° (EK)	Extender Bracer	−56.1 −81.9	82.5 97.5	Strong Controlled Strong Controlled
13	0–27% 27–80% 80–100%	+12.3° −19.8° <10.0°	154.7° (EK)	(Extender) Flexor Bracer	−29.3 +10.2 −3.0	70 40 15	Strong Controlled Mod. Controlled Negligible

## Data Availability

Restrictions apply to the availability of the datasets. The data presented in this study are not publicly available due to ethical and confidentiality restrictions associated with participant consent and institutional approval.
